# Complete mitochondrial genome of *Cyrenobatissa subsulcata* (Veneroida:Corbiculidae)

**DOI:** 10.1080/23802359.2020.1765215

**Published:** 2020-05-14

**Authors:** Xin Liao, Chenghui Liu

**Affiliations:** a Guangxi Mangrove Research Center, Guangxi Key Lab of Mangrove Conservation and Utilization, Beihai, China; b College of Agriculture, Guangxi University, Nanning, China

**Keywords:** Mitochondrial genome, Corbiculidae, phylogeny

## Abstract

In this study, we assembled one mitochondrial genome of *Cyrenobatissa subsulcata* from NovaSeq 6000 sequencing data. The complete mitochondrial genome of *C. subsulcata* was 18,074 bp in length, including 37 genes, which are 13 protein-coding genes, 22 transfer RNA genes, and two ribosomal RNA genes ((12S and 16S), which was similar as another Corbiculidae species, *Corbicula fluminea*. The overall base composition was 26.08% for A, 42.9% for T, 9.11% for C, and 21.91% for G. A phylogeny of 14 bivalves showed *C. subsulcata* was clustered within Corbiculidae.

*Cyrenobatissa subsulcata* (Clessin, 1878) with a local name ‘Big Clam’ is a species of Cyrenobatissa, Corbiculidae, Veneroida. *Cyrenobatissa subsulcata* is the largest known species of Corbiculidae in the world, with a maximum length of 8 cm and a height of more than 7 cm. The clam is distributed in East and South China Sea (Xie En-yi and Ning 2007). To date, mitochondrial and ribosomal sequences of only two species have been reported for Corbiculidae (NCBI, accessed 2020 April 15), which are *Corbicula fluminea* (MK392334) and *Geloina erosa* (MN849878) (Zhang et al. 2019; Liao et al. 2020). Here we assembled the complete mitochondrial genome of *Cyrenobatissa subsulcata,* which could be helpful for assessing its systematic position.

In this study, voucher specimens were obtained from Lianzhou Bay in Beihai, Guangxi, China (21.59°N, −109.12°E) and were vouchered at Guangxi Mangrove Research Center, China (specimen Accession number CS#1-10). The voucher specimen and extracted DNA were stored at −20 °C in GMRC. Total genomic DNA was obtained from muscle of the individual using the QIAamp DNA Micro Kit (Qiagen, Germany) and sequenced by NovaSeq 6000. The mitogenome was assembled with NOVOPlasty v2.7.0 (Dierckxsens et al. 2017), annotated with MitoZ v2.4 (Meng et al. 2019), and deposited in GenBank with an accession number MN849880.

The complete mitochondrial genome of *Cyrenobatissa subsulcata* was 18,074 bp in length, including 37 genes, which are 13 protein-coding genes, 22 transfer RNA genes, and 2 ribosomal RNA genes ((12S and 16S), and all of them were located on the positive strand, compared with the majority of bivalves. The gene composition is similar with *C. fluminea*, but had different order. Compared with *G. erosa,* the mt genome of *C. subsulcata* had one more protein-coding gene, which is ATP8. The mean length of tRNAs was 64 bp, ranging from 62 bp to 68 bp. The base composition was 26.08% for A, 42.9% for T, 9.11% for C, and 21.91% for G, demonstrating a bias of higher A + T content (68.98%) than the G + C content (31.02%). Most PCGs used TAA and TAG as stop codons, excepted for four genes, ND1, ND2, ND4 and COX1, which had incomplete stop codon T.

Fourteen amino acid sequences were aligned using MAFFT v7.407 (Katoh and Standley 2013) and trimmed with trimAl v1.4.1 (Capella-Gutierrez et al. 2009) with the heuristic method ‘automated1.’ The phylogeny of fourteen sequences including three species from Corbiculidae, ten species from other families of Veneroida, and one Arcoida specie as an outgroup, was reconstructed using IQTREE v1.6.10 (Nguyen et al. 2015) with the partitioning method (Figure 1).

**Figure 1.**
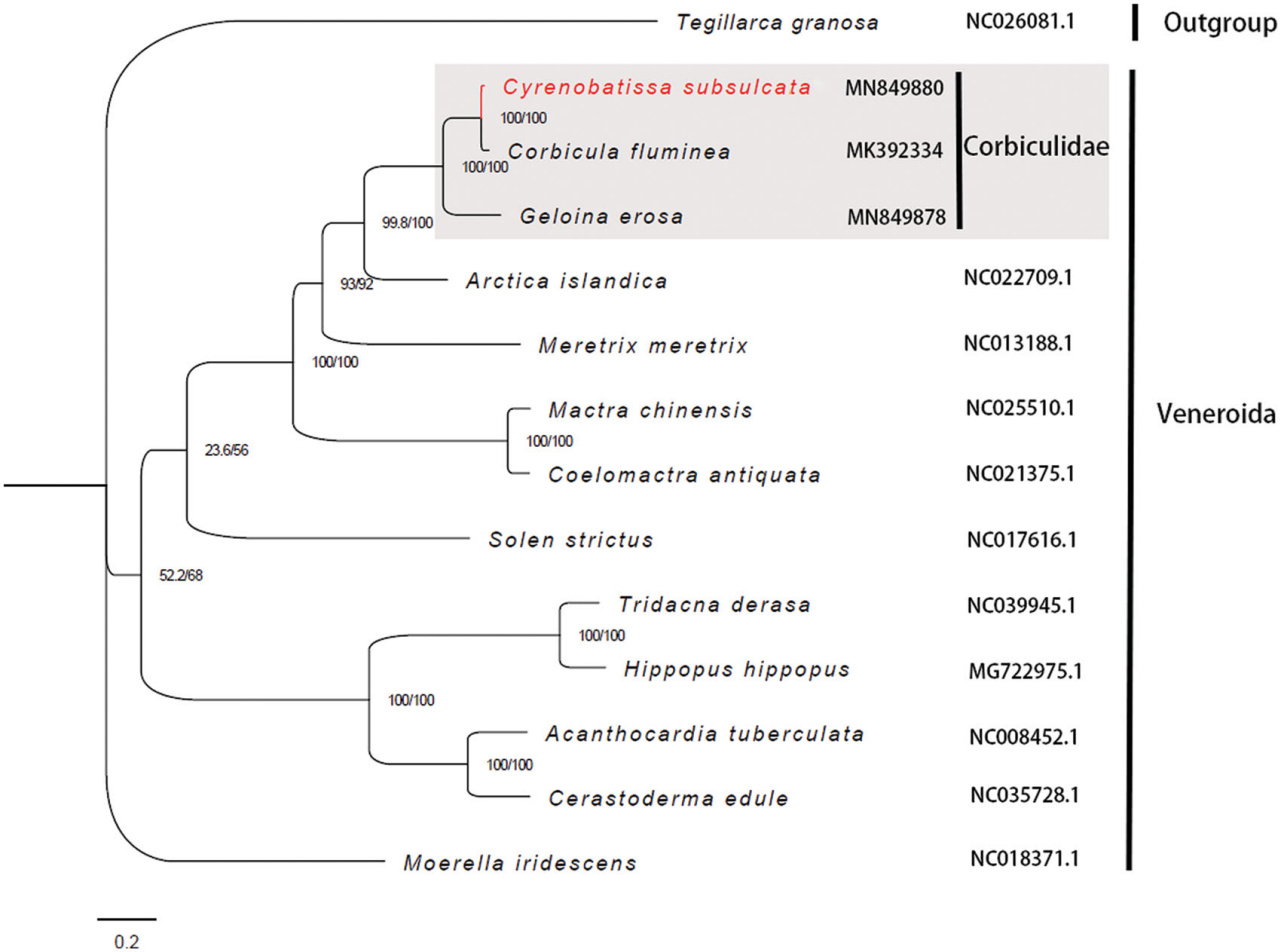
Phylogenetic tree of 13 species from Veneroida and one outgroup using the complete mitochondrial PCGs sequences. The complete mitogenome is downloaded from GenBank and the phylogenic tree is constructed by maximum-likelihood (ML) method. SH-aLRT and UFBoot support values are given on nodes.

## Data Availability

The data that support the findings of this study are openly available in NCBI GenBank database at (https://www.ncbi.nlm.nih.gov) with the accession number is MN849880, which permits unrestricted use, distribution, and reproduction in any medium, provided the original work is properly cited.
